# Perceptions and attitudes about antibiotic resistance in the general public and general practitioners in France

**DOI:** 10.1186/s13756-022-01162-3

**Published:** 2022-10-06

**Authors:** Colette Menard, Sophie Fégueux, Zoë Heritage, Michèle Nion-Huang, Anne Berger-Carbonne, Isabelle Bonmarin

**Affiliations:** 1 Prevention and Health Promotion Direction, Infectious and Environmental Risks Unit, Public Health France, Saint-Maurice, France; 2 Public Health France, Infectious Diseases Direction, Saint-Maurice, France

**Keywords:** Antibiotic resistance, Perceptions, Attitudes, General public, General practitioners

## Abstract

**Background:**

During the last 20 years, France has taken important steps to tackle antibiotic resistance. These include national awareness campaigns for the general public, and supporting changes in terms of antibiotic prescription for healthcare practitioners. To prepare the upcoming 2022/2023 campaign, we conducted two surveys to assess (1) the general public’s knowledge, attitudes and behaviours regarding antibiotics and (2) the perceptions and practices of general practitioners (GPs).

**Methods:**

Two quantitative telephone surveys were conducted using the same methodology as that used in 2010 by the National Health Insurance Authority. The first was conducted in 2019 in a national representative quota sample of 1204 persons aged over 15 years living in metropolitan France, including an over-sample of 332 parents of children aged six years or under. The second was conducted in 2020 in a national representative sample of 388 GPs.

**Results:**

Twenty-seven percent of respondents reported taking antibiotics during the previous year. Sixty-five percent of GPs declared prescribing fewer antibiotics during the previous five years. However, 33% of GPs reported they often had patients who put high pressure to get antibiotics. The pressure from elderly patients, especially those with comorbidities was notable. Three percent of respondent patients reported putting often pressure on their GP. All respondents expressed total trust in their GP irrespective of whether s/he had prescribed them antibiotics. Half knew that antibiotics act only on bacteria, and 38% said they understood precisely what antibiotic resistance is.

**Conclusion:**

Although antibiotic use is decreasing in France, patient pressure on GPs to prescribe antibiotics is very high. GPs are key ambassadors in reducing antibiotic use. Awareness campaigns must target elderly patients in particular.

**Supplementary Information:**

The online version contains supplementary material available at 10.1186/s13756-022-01162-3.

## Introduction

Despite an 18% drop in the number of antibiotic prescriptions for non-hospitalised patients between 2009 and 2019 [1], France remains one of the countries with the highest antibiotic consumption in Europe [2]. Since the early 2000s, several national action plans have been implemented to tackle antibiotic resistance [3–5]. The two major approaches in these plans are (1) increasing public awareness, and (2) informing and training health professionals.

With regard to public awareness, campaigns to date have been organised by the Ministry of Health and the National Health Insurance Authority. The first such campaign, called “*Les antibiotiques, c’est pas automatique” (“antibiotics are not automatic”),* ran from 2002 to 2005, and was associated with a 26.5% decrease in total antibiotic use [6, 7]. The two following campaigns, continued through 2012, were less successful.

French health authorities have supported changes for antibiotic prescription by healthcare professionals via: (1) regular awareness raising for general practitioners (GPs) through visits from Health Insurance representatives [8], (2) personalized information enabling them to compare their prescription practice to that of their colleagues, (3) support in using an antibiotic non-prescription pad [9], (4) an annual allowance based on quality indicators including antibiotic prescription [10, 11], (5) best practice recommendations and tools [12] and (6) support in using rapid streptococcal throat tests (RST).

Santé publique France, the French national public health agency, is responsible for monitoring antibiotic use and resistance in the country. It will organise future nationwide awareness campaigns. To establish the upcoming campaign, we needed to assess the knowledge and practices of the French population and their GPs regarding antibiotics.

## Methods

### Survey design and recruitment

We organized two quantitative surveys in 2019 and 2020, using the same methodology as that used in 2010 when the National Health Insurance ran related surveys. The same Institute, BVA group, collected data.

The first survey was conducted in October 2019 and comprised a telephone survey of a representative sample of the general public aged over 15 years, residing in metropolitan France. Samples were constructed using quotas based on the following variables: gender, age, social category of the household reference person, region and urban area type. Quotas came from the 2015 general population census data. A total of 1,204 persons were interviewed, including an over-sample of 332 parents of children aged six years or under (see Additional file 1: Fig. S1). The second survey was conducted in March 2020 and comprised a telephone survey of a national representative sample of 388 GPs. Only private practice doctors and those mixing private practice with hospital-based work were included. The representative sample was drawn from a panel of 6,000 GPs previously recruited by the BVA group for other surveys. Representativeness was ensured by sampling quotas using the following variables: gender, age, region and type of practice (2018 reference data from the French Ministry of Health). Respondents received a small payment for participating in the study (See Additional file 1: Fig. S2).

### Survey content

The general public survey [1] explored participants’ consumption of antibiotics during the previous 12 months, their demand that they be prescribed antibiotics, and their knowledge and beliefs about antibiotic resistance.

The GP survey explored doctors’ perception of the evolution of their prescription of antibiotics during the previous five years, their current antibiotic prescription practices, their attitudes towards patients’ demands for antibiotics, their use of various diagnostic tests and prescribing tools (RST, C-Reactive Protein level (CRP), applications, websites), and their perception of previous antibiotic awareness campaigns.

The questions in the general public and GP surveys were identical to those in the 2010 surveys. However, the 2019 and 2020 surveys also contained extra items on new tools and sources, such as the use of non-prescription pads and smartphone applications. Questions were a yes or no answers or with possible answers being: ‘Yes, often’/‘Yes, sometimes’/’Yes, rarely’/’No, never’/’Don’t know’ or ‘Totally agree’/’Mostly agree’/‘Mostly disagree’/’Totally disagree’.

### Survey analysis

A descriptive analysis using Student’s t-test was performed for surveys using socio-demographic variables. As we did not have the 2010 surveys database, statistical comparisons were not systematically done and we only mentioned in the discussion those provided by BVA or those we were able to calculate.

## Results

### Reported antibiotic use

Twenty-seven percent of the study population reported receiving antibiotics for themselves during the previous twelve months. Just over half (54%) of those who had a child aged six years or under reported a prescription for antibiotics for their child(ren) during the previous year.

**Fig. 1:**
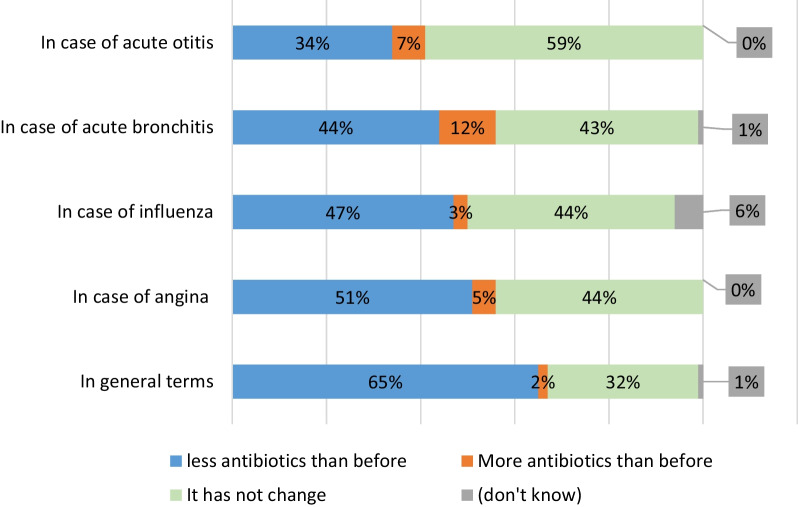
Evolution of GP prescriptions in the previous five years. *Do you feel that you have prescribed fewer antibiotics, more antibiotics, or the same amount of antibiotics over the last five years?*

With regard to the GP survey, 65% declared they had reduced their prescriptions for antibiotics over the previous five years. According to the disease, it varied from 51% reduction for angina to 34% for acute otitis. Only 2% reported an increase in antibiotic prescription (Fig. 1).

### Patients pressure

**Fig. 2:**
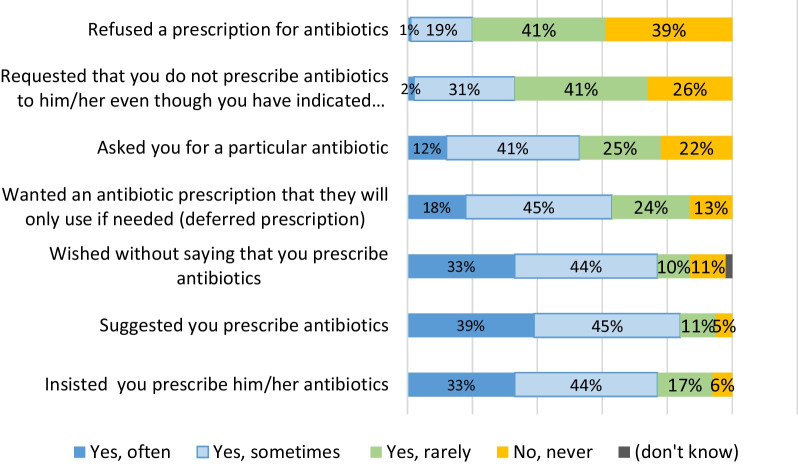
Patient pressure for antibiotics: Perception of GPs. *Have you ever had a patient with a disease or infection who…*

**Fig. 3:**
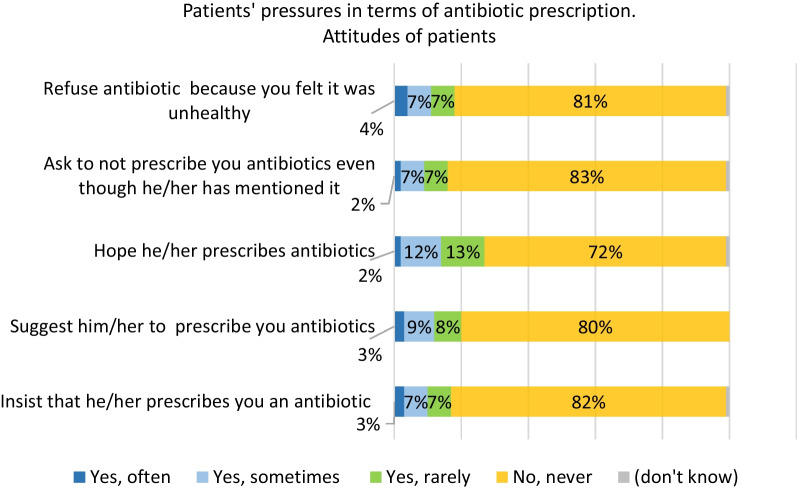
Patients’ pressure for antibiotics. *In the past 12 months, have you personally ever visited a doctor with an infection or illness and…*

Among GPs, 33% reported having often patients who insisted on having antibiotics (Fig. 2) when only 3% of participants said they did (Fig. 3). There is almost the same discrepancy regarding patients who just suggest on having antibiotics: 39% of GPS have often this perception and 3% of participants reported they did it often. In contrast, only 6% of GPs reported not having pressure from their patients for an antibiotic prescription.

**Fig. 4:**
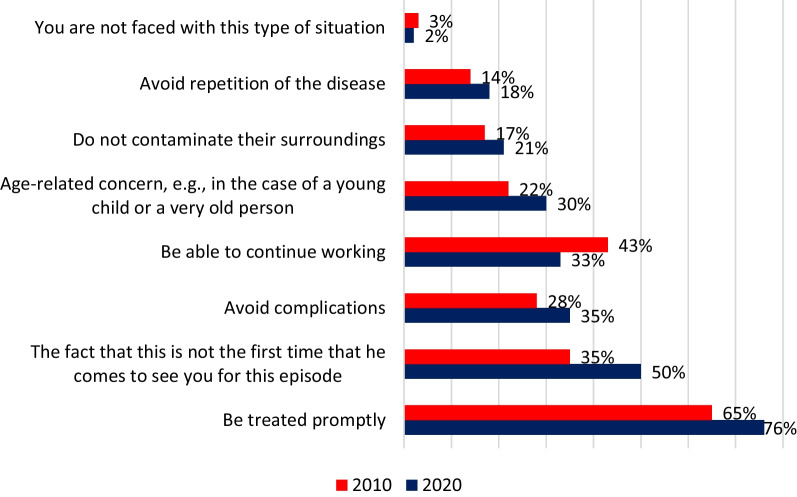
Patients’ reasons for wanting a prescription, according to GPs

GPs reported that the most common reasons given by patients demanding antibiotics were to treat the infection promptly (76%), and because they had already had a consultation for the illness (50%) (Fig. 4).

**Fig. 5:**
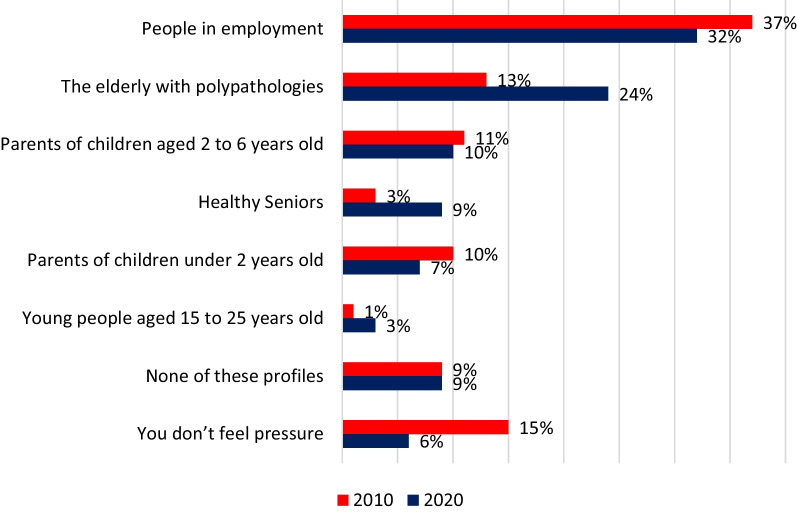
Patients most in demand of antibiotics, according to GPs

In 2020, GPs reported that people in employment were the category most frequently requesting antibiotics (32%), followed by the elderly with multiple pathologies (24%) (Fig. 5).

### GPs’ prescription practices and patients’ trust

When a patient insisted, only 2% GPs reported giving in and prescribing antibiotics directly even though the antibiotics were not useful.

The majority of GPs (64%) declared they did not automatically prescribe antibiotics but advised the patient to contact them again in two or three days if symptoms persisted. Thirty-two percent of participants in the general public survey reported they had experienced this situation.

Thirty-four percent of GPs declared using different strategies to delay prescribing antibiotic (e.g., prescribing antibiotics to be taken only if symptoms persisted or worsened). Twenty-four percent of participants in the general public survey reported having be faced to this practice.

The public expressed a high level of trust in their GP’s diagnosis irrespective of whether s/he prescribed antibiotics (89%) or not (91%). Only 14% of patients thought that non-prescription meant the doctor took their illness lightly (Table 1).

**Table 1:** General public survey participants’ perception of their GP in case of prescription or non-prescription

When a GP prescribes antibiotics, I usually think (n = 584)		When a GP does not prescribe antibiotics, I usually think (n = 620)	
He/she is a professional who can make the right diagnosis	89%	91%	He/she is a professional who can make the right diagnosis
He/she considers that antibiotics will be effective against my disease	86%	75%	He/she considers that antibiotics will not be effective against my disease
He/she takes my illness seriously	80%	14%	He/she takes my illness lightly
He/she does not want me to come back to him/her in a few days (if my condition does not improve)	37%	66%	He/she considers that at the first stage of disease development, antibiotics are not justified

Base: The following two situation were put to half of the general public sample (% totally and rather agree)

### The use of diagnostic tests and antibiotic stewardship tools

**Fig. 6:**
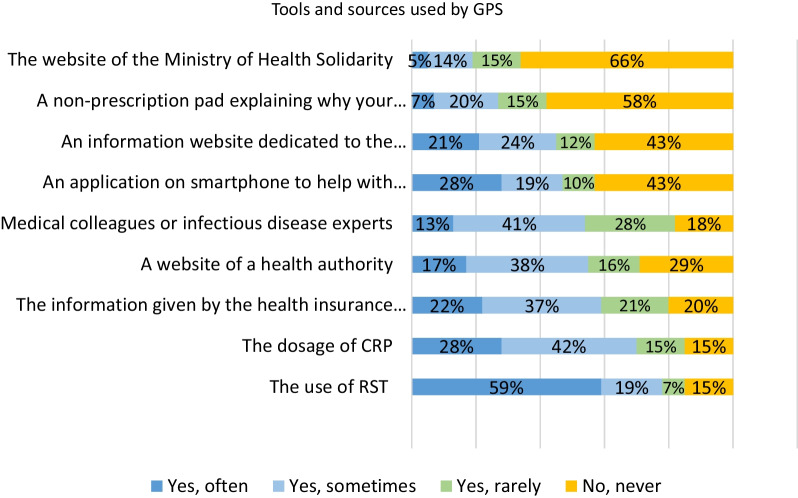
Tools and sources used by GPs. *Do you use the following information sources or tools?*

Eighty-five percent of GPs reported having used RST and 59% used them often (Fig. 6), RST were perceived as useful for reducing diagnostic uncertainty, helpful to justify to patients whether or not they should receive a prescription. They were also considered an educational tool to help patients better understand the origin of their illness and the recommended treatment. Furthermore, 85% of GPs had used CRP, and 42% a non-prescription pad explaining why antibiotics are not necessary. In contrast, 12% of patients reported receiving this non-prescription pad.

Applications (e.g., Antibioclic [13]) and websites dedicated to antibiotics (e.g., Antibio'Malin, antibioEst [14, 15] etc.) were used by a majority of GPs (57%). GPs under 40 years old were more likely to have “often or sometimes” used stewardship tools such as applications and dedicated sites.

### Public and GP understanding of antibiotic resistance

During the previous 12 months, 9 percent of GPs reported had often come across cases of antibiotic resistance.

**Fig. 7:**
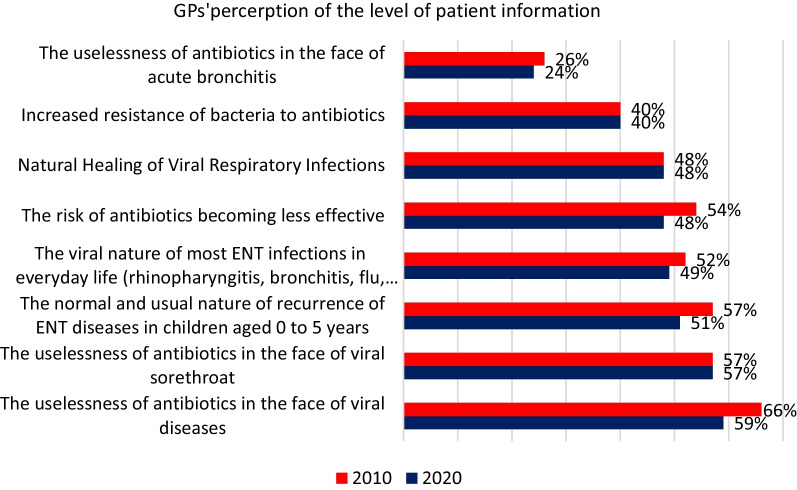
GPs’ perception of patients’ level of information about antibiotics. *Would you say that your patients seem to be well informed about the following (% Yes)*

In 2020, 59% of GPs believed that their patients were well informed about the ineffectiveness of antibiotics against viruses. This proportion dropped to 24% regarding their knowledge about the ineffectiveness of antibiotics for acute bronchitis (Fig. 7).

In the general public survey, 88% participants felt well informed about the need to strictly comply with medical prescriptions. More specifically, 96% knew that they should not take a double dose of antibiotics if a dose is missed, 88% were aware that leftover antibiotics from a previous treatment should not be reused, and 82% that antibiotic treatment should not be interrupted even when one feels better. Sixty percent had already heard about antibiotic resistance. However, only 38% of all participants and 38% of parents with a child aged six years or under indicated that they knew exactly what it was. Low social economic status workers and the elderly were the least informed.

### Persisting misconceptions

**Fig. 8:**
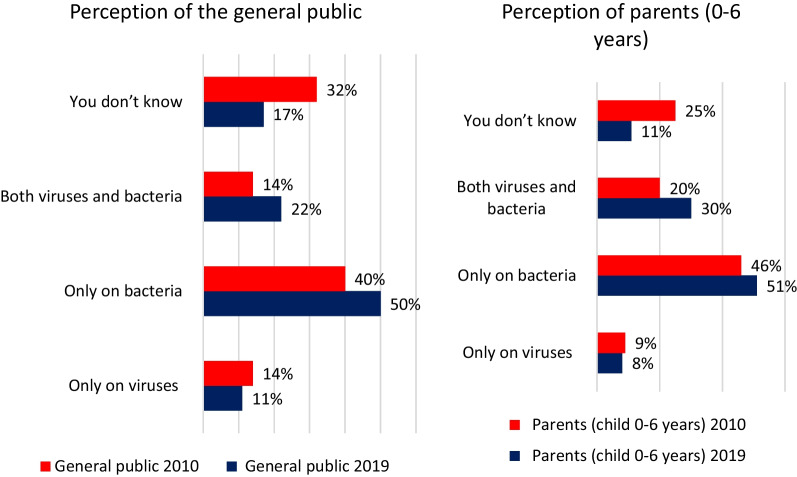
General public and parents’ with children =  < six years old perceptions of how antibiotics work. *Do you think antibiotics work…*

Only half the participants in the general public survey knew that antibiotics only target bacteria (Fig. 8).

Even if parents of young children have globally a better knowledge, less than half of the respondents in the general public survey could not individuate for which diseases antibiotics are or are not effective (Table 2).

**Table 2:** General public and parents with children =  < six years old knowledge about the effectiveness of antibiotics on diseases (% who gave the correct answer)

	All	Parents of children = < 6 years old	General public (with no child = < 6 years old)
*Sample size*	*n* = *1204*	*n* = *332*	*n* = *872*
Gastroenteritis (not effective)	50%	61%**	**49%**
Ear infection (in some cases)	49%	53%	48%
Bacterial sore throat (effective)	46%	57%**	**45%**
Influenza (not effective)	43%	46%	43%
Sinusitis (in some cases)	40%	37%	40%
Pneumonia (in some cases)	39%	40%	39%
Viral sore throat (not effective)	38%	39%	38%
Urinary tract infection (effective)	36%	42%*	**35%**
Common cold (not effective)	36%	34%	36%
Bronchiolitis (not effective)	21%	25%	21%
Acute bronchitis (not effective)	15%	21%*	**15%**

Bold values indicates that significantly better knowledge among parents than the general public

*p < 0.05 **p < 0.01

Half believed that antibiotics always helped a person to get well quickly and saved time, and 44% believed that antibiotics helped workers to become fit enough to return to work quickly.

In most cases, young people aged 15 to 24 years old, people older than 64 years old, and the lowest socio-professional categories were the least well informed. Older people were the sub-population with the most inaccurate beliefs about antibiotics.

### Information campaigns seen as a useful communication tool

Eighty-nine percent of GPs thought that public awareness campaigns helped to support their explanations to patients regarding antibiotics, and 87% believed these campaigns increased patient acceptance of their decision not to prescribe them. Seventy-seven percent reported that campaigns helped to reduce the pressure patients put on them to obtain a prescription. Most of the GPs (60%) felt campaigns helped save time during consultations.

One in two GPs (55%) reported changing the way they prescribed antibiotics as a result of awareness campaigns. Physicians aged over 60 were particularly likely to feel that campaigns supported their practice (85%), and had changed the way they prescribed (62%).

## Discussion

The two surveys we conducted in 2019 and 2020 aimed to assess the general public’s and GPs perceptions about antibiotic use, antibiotic resistance, and the perceived effectiveness of awareness-raising campaigns.

Even if it was almost stable for the public (27% versus 31% in 2010), our findings show a decrease over time in the children’s use of antibiotics during the last twelve months: 54% of parents said their child(ren) aged 6 years or under had received an antibiotic, compared to 65% from 2010. During the last five years, 65% of GPs declared having reduced their prescriptions for antibiotics in general. Only 2% stated they prescribe more.

This is in line with sources that find a reduction of the use of antibiotics in France [1, 3–5, 10, 11]. Between 2009 and 2019, the number of antibiotic prescriptions in France decreased by 18% among all age groups, except the elderly [1]. However, difficulties in limiting prescriptions to treat otitis, influenza and acute bronchitis should be noted. These three illnesses account for 40% of all antibiotic prescriptions [10].

In line with previous findings in the US [16], the UK [17] and France [6], 33% of our surveyed GPs reported often high pressure from their patients to prescribe antibiotics. Only 3% of patients declared insisting on their GP “often” for an antibiotic. This reflects the divergence in perception between patients and GPs observed for several common questions. It could also be related to reporting bias: difficulty for patients to admit that they are pressuring and justification for some physicians for overprescribing antibiotics.

Compared to the 2010 survey, where the "often" and "sometimes" responses were mixed, GPs felt greater pressure from patients insisting on a prescription (69% in 2010 vs. 77% in 2020). According to GPs, the percentage of elderly with comorbidities increased among the patients most in demand of antibiotic (13% in 2010 vs 24% in 2020). Among the public, 17% reported they had put some «pressure» on their GP often or sometimes (13% in 2010).

However, nearly all the participants in the general public survey (90%) declared that they trusted their GP’s decision irrespective of whether s/he prescribed them antibiotics or not. This finding is very important to reassure GP, and highlights that they are important ambassadors for antibiotic stewardship [6, 18, 19].

Only 2% of physicians report prescribing antibiotics when patients insist. This proportion may be underestimated. Nevertheless, many physicians are adopting strategies to avoid systematic prescribing. Two thirds advised their patients to wait several days without a prescription of antibiotics in case of a suspected viral infection, and a third used delayed prescribing strategies. Less than half (42%) use the non-prescription pad that explains to the patient why he or she will not receive antibiotics. Only 12% of patients seemed to be aware of this tool. Beyond the reporting bias and the perception discrepancy mentioned above, these results show that its use should be developed.

The majority of GPs used RST, CRP and digital decision support tools, such as smartphone applications and dedicated websites, were used to a lesser extent. GPs reported that these digital tools facilitated consultations and their communication with patients, especially those requesting explanations and/or insisting that antibiotics be prescribed [15].

Finally, GPs were very favourable of national public awareness campaigns and considered the could help them to reduce antibiotic prescription demands. A high percentage of participants in the general public survey felt they were well informed about antibiotic use and compliance with prescriptions. But despite an improvement, the proportion of people saying they know what is antibioresistance is only 38% in 2019 (29% in 2010) and only 50% of the participant’s knew that antibiotics are only effective against bacteria (40% in 2010).

In line with Eurobarometer findings [20] and recent qualitative research in France [6], we found that misconceptions about antibiotics persisted. Large gaps in knowledge remained about the effectiveness of antibiotics for several diseases [21, 22].Young people, the elderly and persons from the lowest social economic status categories were the least informed populations.

## Strengths and limitations

The two quantitative studies described here complement qualitative studies also conducted in France [6, 15]. They are particularly useful to obtain a representative overview of the general public’s and GPs’ perceptions of antibiotic prescription ten years after the previous surveys. Unfortunately, the 2010 results have never been published and we did not have all the database for a systematic statistical comparison. However, we presented in the discussion the comparison when the differences were statically significant.

Self-report surveys may not reflect real behavior or practices. However (i) panels from which the study samples were drawn are regularly renewed; (ii) recall and social desirability biases were the same for the 2010 and 2019/2020 surveys; (iii) results were in line with antibiotic prescription data and other similar studies. Moreover, GPs perceptions cannot be generalized to all healthcare professionals who prescribe antibiotics.

The GPs survey started 2 weeks after the first Covid-19 lockdown. Since the questions were related to the past 12 months, we do not believe that Covid-19 may have had a major impact on the results.

## Conclusion

Although antibiotic use is decreasing in France, patient pressure on GPs to prescribe antibiotics is very high. The French population still only has partial knowledge of antibiotic resistance and needs to be better informed especially elderly people. GPs are key ambassadors in reducing antibiotic use. A new public information campaign will take in account results of our survey in 2022–2023. It will be preceded by support and information for health professionals.

## Supplementary Information

**Additional file 1**. Fig. S1 General Public Sample Structure. (% Adjusted) and Fig. S2 General Practitioners’ Sample structure

## Data Availability

The surveys generated in 2010 have not been published. All questionnaires are available from the authors on request.

## Abbreviations

GPs: General practitioners
RST: Rapid streptococcal throat tests
CRP: Reactive Protein level
