# Supplementary material for: Perceptions and attitudes about antibiotic resistance in the general public and general practitioners in France
Source: Antimicrob Resist Infect Control. 2022 Oct 6;11:124. doi: 10.1186/s13756-022-01162-3 (PMC9536052; doi:10.1186/s13756-022-01162-3)
Supplement: Supplementary file 1 — Additional file 1. Fig. S1 General Public Sample Structure. (% Adjusted) and Fig. S2 General Practitioners’ Sample structure [file 13756_2022_1162_MOESM1_ESM.docx]

**Additional files**

| Fig S1. General Public Sample Structure. (% Adjusted) | | |  |
| --- | --- | --- | --- |
|  |  |  |  |
|  | **TOTAL** | **Parents of child(ren) 0-6 years old** |  |
|  |  |  |  |
| TOTAL | 100% |  |  |
|  |  |  |  |
| Parents with child(ren) 0 to 6 years old | 14% |  |  |
|  |  |  |  |
| Persons with no child 0 to 6 years old | 86% |  |  |
|  |  |  |  |
| *GENDER* |  |  |  |
| Men | 48% |  |  |
|  |  |  |  |
| Women | 52% |  |  |
|  |  |  |  |
| *AGE (years)* |  |  |  |
| 15-24 | 14% | 4% |  |
|  |  |  |  |
| 25-34 | 15% | 44% |  |
|  |  |  |  |
| 35-49 | 25% | 49% |  |
|  |  |  |  |
| 50-64 | 24% | 2% |  |
|  |  |  |  |
| 65 and over | 22% | 0% |  |
|  |  |  |  |
| *OCCUPATION OF HEAD OF HOUSEHOLD* |  |  |  |
| Self-employed and entrepreneurs | 7% | 9% |  |
|  |  |  |  |
| Executives and higher intellectual professions | 13% | 18% |  |
|  |  |  |  |
| Intermediate professions | 15% | 22% |  |
|  |  |  |  |
| Employees | 11% | 15% |  |
|  |  |  |  |
| Manuel Workers | 18% | 31% |  |
|  |  |  |  |
| Retired | 30% | 1% |  |
|  |  |  |  |
| Other inactive | 5% | 4% |  |
|  |  |  |  |
| *REGION* |  |  |  |
| Ile de France | 19% | 21% |  |
|  |  |  |  |
| Paris Basin West | 9% | 9% |  |
|  |  |  |  |
| Paris Basin East | 8% | 8% |  |
| West | 14% | 13% |  |
|  |  |  |  |
| North | 6% | 7% |  |
|  |  |  |  |
| East | 8% | 8% |  |
|  |  |  |  |
| Center East | 12% | 12% |  |
|  |  |  |  |
| Sud West | 11% | 10% |  |
|  |  |  |  |
|  |  |  |  |
| Mediterranean region | 13% | 12% |  |
|  |  |  |  |
| *SIZE OF AGGLOMERATION* |  |  |  |
| Rural area | 23% | 24% |  |
|  |  |  |  |
| Fewer than 20,000 inhabitants | 17% | 17% |  |
|  |  |  |  |
| 20,000 to 100,000 inhabitants | 14% | 12% |  |
|  |  |  |  |
| More than 100,000 inhabitants | 30% | 29% |  |
|  |  |  |  |
| Paris agglomeration | 16% | 19% |  |
|  |  |  |  |

| Fig S2. General Practitioners sample structure. | |
| --- | --- |
|  |  |
| *TYPE OF ACTIVITY* |  |
| Exclusive private practice | 88% |
| Mixed activity (private practice and hospital) | 12% |
| *GENDER* |  |
| Men | 59% |
| Women | 41% |
| *AGE (years)* |  |
| under 40 | 22% |
| 40-49 | 17% |
| 50-59 | 29% |
| 60 and over | 32% |
| *REGION* |  |
| Grand Est | 8% |
| Nouvelle-Aquitaine | 10% |
| Auvergne-Rhône-Alpes | 12% |
| Normandy | 5% |
| Bourgogne-Franche-Comté | 4% |
| Brittany | 5% |
| Centre-Val de Loire | 3% |
| Ile-de-France | 15% |
| Occitanie | 10% |
| Hauts-de-France | 9% |
| Pays de la Loire | 6% |
| Provence-Alpes-Côte d'Azur, Corse | 10% |
| DOM/TOM | 3% |
